# Address

**Published:** 1904-10-15

**Authors:** Charles Godon

**Affiliations:** Paris, France


### ADDRESS.

BY DR. CHARLES GODON, PARIS, FRANCE.

President of the International Dental Federation.

MR. PRESIDENT, LADIES AND GENTLEMEN:—The first thought I wish to express here, in the name of the F. D. I., is a thought of admiration for your great nation, your mighty republic to which are bound by so many ties of sympathy and gratefulness all the citizens of the world who are fond of liberty, progress and civilization.

Your magnificent exhibition is a new proof of the progressive genius of the American nation and deserves the first place among the manifestations of human activity. Hail to the splendid city of St. Louis, the place of it, and thanks for the hearty welcome to us.

I thank you, Mr. Rogers, for doing us the great honor of presiding over this first meeting. And hail heartily to the American dental profession to which modern dentistry owes its most important progress, and whose most eminent representative members are here with us. Hail to the members of the dental profession of the world who have answered our calling and among them the members of the Executive Council, and all who have taken an active part in the evolution of this great Congress.

Today we inaugurate in St. Louis the session that finishes the work of the first period of the F. D. I. We shall soon have to account to the Congress of 1904 for the mandate trusted to us by the Congress of 1900 and give them back our powers. I shall not make a long report of the work of the F. D. I. during that period of four years; it is the task of our General Secretary and devoted co-laborer, Dr. E. Sauvez, who has participated in our work from the beginning. I only wish to recall to you the principal phases through which the F. D. I. has passed.

Born in Paris in 1900 from the enthusiasm of the twelve hundred delegates from every part of the world, it uttered its first lispings in Cambridge the following year, in the old English University, under the presidency of the eminent professor of physiology, Sir Michael Foster, who determined the real philosophical formula, defining and limiting the action of the F. D. I. in order to make of it a great advisory council of the world's dentistry.

Then next in Stockholm took place the session where in the midst of discussions for the working out of an international curriculum of education and the determination of the best conditions for the most effective public dental services, was decided the meeting in St. Louis of the Fourth International Dental Congress, in which we shall participate next week. From that decision ensued a long, feverish and fruitful contention about the interpretation to be given to the powers of the Federation or its representative members and relative to the conditions of its intervention in the different kinds of professional manifestations; that discussion was long, as it lasted for more than two years, and the session of Madrid in 1903 was almost exclusively devoted to it; feverish, for it has caused deep discussions in the professional body, especially in the United States, and fruitful also for thus it has manifested its existence to every one, the importance of its action in the proceedings of worldly professional life and come to such a perfection of its constitution and rules that when confirmed by the next Congress they will secure for a long time, I hope, the success of succeeding periods.

A cedar requires more time to grow than a reed, says the poet, but lasts longer. So during those four years the F. D. I. has grown slowly, spreading its roots in deeper and deeper layers of the professional life of every country, covering worldly dental manifestations with its sheltering branches, wider and greater and more and more numerous.

So it has proved the necessity of its existence by the acts it has performed during those four years, for the plan of work we leave is large enough to occupy several sessions, viz: International Bibliography, Nomenclature, Code of Ethics, etc. So it will continue growing and the future will enlarge, I hope the work that has already been done. And should it perish after this first period, we should have the consciousness of having performed good work by so sowing all over the world just and useful ideas in favor of human progress.

In our twentieth century these international federations constitute a new organism, not only necessary to worldly dentistry, but to all departments of professional and scientific activity. When men thus meet in great peaceful sessions as universal exhibitions and congresses, wholly given up to the pleasure of exchanging their ideas, of communicating the result of their works and researches to each other, they must forget political and geographical limits and think only of drawing nearer to one another in those great international federations which are constituted in every branch of human activity and which now meet regularly at different points of the world.

As new agents of progress and civilization, these great federations do not attack the essential part of nationality, for that remains under the safe-keeping of political and diplomatic bodies, but their activities take place on the high plane of human science where territorial divisions have no action, where the end of strife can but be pacific, because the contests are only of the intellect, where the presiding goddess is like the splendid statue of Liberty in the harbor of New York, the torch of Truth in one hand, and in the other the olive branch, symbols of true human fraternity.

